# Complete genome sequence of *Robbsia andropogonis* strain FG-1 causing bacterial leaf spot disease on *Pueraria montana* var. *thomsonii* in China

**DOI:** 10.1128/mra.00674-25

**Published:** 2025-09-29

**Authors:** Xiaoxue Wu, Xiaodong Cui, Hong Liu, Tao Xie, Lixia Zheng, Wensheng Chen, Tao Li

**Affiliations:** 1 Department of Horticulture, Foshan University 47868, Foshan, China; 2 Independent Researcher, Guangzhou, China; The University of Arizona, Tucson, Arizona, USA

**Keywords:** *Robbsia andropogonis*, Pueraria montana var. thomsonii, bacteria, complete genome

## Abstract

This study presents a complete genome of *Robbsia andropogonis* strain FG-1, the causative agent of bacterial leaf spot disease on *Pueraria montana* var. *thomsonii* in China. Hybrid long- and short-read sequencing yielded a high-quality assembly, providing essential data for investigating virulence mechanisms and developing control strategies.

## ANNOUNCEMENT

**Fig 1:**
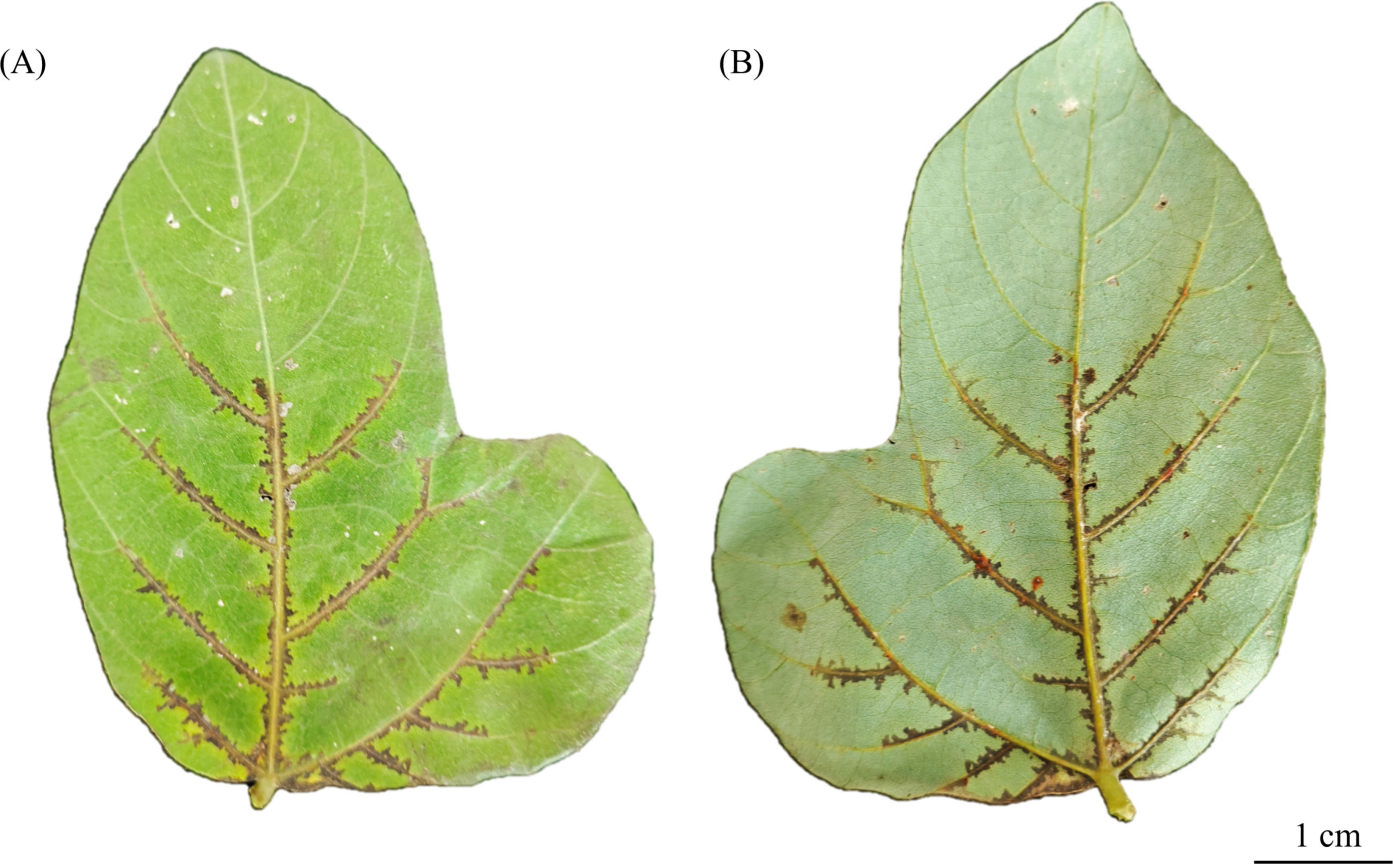
Symptoms induced by *R. andropogonis* strain FG-1 on *Pmt* leaves 7 days post-inoculation. (**A**) Adaxial leaf surface showing radiating water-soaked lesions with chlorotic haloes (scale bar = 1 cm). (**B**) Corresponding abaxial surface exhibiting necrotic centers and translucent margins.

*Robbsia andropogonis* is a gram-negative plant pathogen causing bacterial leaf spot disease (1). In China, *R. andropogonis* has recently caused significant economic losses to *Pueraria montana* var. *thomsonii* (*Pmt*) cultivation in Guangdong Province (2). This study reports the complete genome sequence of *R. andropogonis* strain FG-1 from *Pmt*, facilitating deeper insights into the pathogen’s virulence mechanisms and informing future disease control strategies. Strain FG-1 was isolated in August 2024 from a *Pmt* leaf showing characteristic bacterial leaf spot symptoms, collected from Gaoming District, Guangdong Province, China (22.53N, 112.53E). Following surface sterilization with 75% ethanol and triple rinsing with sterile water, tissues from the disease-health junction were macerated in sterile water. The resulting suspension was streaked onto LB culture medium and incubated at 30°C in darkness for 3–5 days. Pure cultures were obtained through at least three successive rounds of subculturing individual colonies exhibiting characteristic morphology: gray-white to dull white, mucoid, convex, circular, and smooth (2). Pathogenicity was confirmed by spray-inoculating young leaves of a 3-month-old *Pmt* plant with bacterial suspension (10⁸ CFU/mL). Leaves were individually bagged with fresh-keeping bags for 24 h at 100% RH under 30°C/16 h light and 22°C/8 h dark cycles. Characteristic water-soaked lesions with yellow haloes developed interveinally within 7 days, matching field symptoms (Fig. 1). Koch’s postulates were fulfilled through pathogen reisolation from lesions. A single colony was inoculated into 50 mL of LB broth and incubated at 30°C for 12 h. Genomic DNA was extracted from the bacterial culture using E.Z.N.A. Bacterial DNA Kit, DNA from a single extraction was used for both libraries. The identity of the isolate was confirmed as *R. andropogonis* by Conventional PCR amplification of the full-length 16S rRNA gene using universal bacterial primers 27F/1492R and by amplifying the expected 704 bp product using species-specific primers LJ23f/LJ24R (3, 4). Sequencing was performed using a combination of long-read and short-read technologies. Long-read data was generated using the Oxford Nanopore Technology PromethION platform with the SQK-RBK110.96 kit on a FLO-PRO114M flow cell, yielding raw sequencing data. After quality control, this resulted in 688,274,112 bp of Nanopore data from 395,669 reads, with a mean read length of 1,739.5 bp, an N50 of 3,224 bp, and a maximum read length of 163,189 bp. Short-read data were generated using the Illumina HiSeq platform (SBS technology), with libraries prepared using the Nextera XT kit for 2×150 bp paired-end sequencing. Following quality control, 13,397,406 clean reads (2,004,581,918 bp) were obtained, providing 333× coverage. Default parameters were used for all software unless otherwise specified. A hybrid assembly was generated using Unicycler v0.4.9 with Illumina reads for contigs and Nanopore reads for scaffolding (5). The assembly underwent two rounds of error correction with Pilon v1.23 (6), correcting 37 insertions and 75 deletions in Round 1, and 38 SNPs in Round 2. The final assembly comprised eight contigs with an overall GC content of 59.00% and a total size of 6,011,680 bp, consisting of three circular chromosomes (3,612,886 bp, 1,112,911 bp, and 557,219 bp) and five circular plasmids (178,005 bp, 153,059 bp, 138,375 bp, 130,044 bp, and 129,181 bp). Genome completeness and quality were assessed using CheckM v1.2.3, BUSCO v6.0.0 (lineage data set: burkholderiaceae_odb12, mode: prok_genome_prod), and through annotation by the NCBI Prokaryotic Genome Annotation Pipeline (PGAP), revealing a completeness of 92.02% (CheckM) and 97.00% (BUSCO), contamination of 0.82%, and an Average Nucleotide Identity of 99.32% against *R. andropogonis* type strain BLB1 (GenBank GCA_034047095.1) (7–9). PGAP annotation predicted 5,652 genes in total, comprising 5,033 protein-coding genes, 548 pseudogenes, 55 tRNA genes, 12 rRNA genes, and 4 ncRNA genes.

## Data Availability

The raw sequence data and assembled genome generated in this study have been deposited in public repositories. The BioProject accession number is PRJNA1260466, and the BioSample accession is SAMN48396344. Long-read sequencing data are available under the Sequence Read Archive (SRA) accession SRR34063158, while short-read sequencing data are accessible under SRA accession SRR34063159. The complete annotated genome sequence has been assigned GenBank accession numbers CP191271.1 to CP191278.1.
